# Incidence of Anterior Cruciate Ligament Injury and Subgroup Analysis by Age, Sex, Injury Mechanism, and Severity Among Bangladeshi 1853 Soccer Players: A 1‐Year Prospective Cohort Study

**DOI:** 10.1002/hsr2.72889

**Published:** 2026-07-25

**Authors:** Sohraf Hossain, Kazi Md. Azman Hossain, Md. Shafiqul Islam, Md. Adnan Ibne Reza, Tamzid Hossain, Md. Nazmul Haq, Kulsum Akter

**Affiliations:** ^1^ Department of Physiotherapy Centre for the Rehabilitation of the Paralysed (CRP) Savar Bangladesh; ^2^ Department of Physiotherapy and Rehabilitation Jashore University of Science and Technology (JUST) Jashore Bangladesh

**Keywords:** ACL injury, cohort, sex, soccer

## Abstract

**Background and Aims:**

Anterior cruciate ligament (ACL) injuries are a major concern in soccer due to their high incidence and long‐term functional impact. Despite this, there is a lack of prospective epidemiological data on ACL injuries among soccer players. This study aimed to investigate the incidence of ACL injuries among Bangladeshi soccer players during a 1‐year follow‐up period, with subgroup analyses by age, sex, injury mechanism, and severity.

**Methods:**

A 1‐year prospective study was conducted involving 1853 participants during the 2024–2025 session across 12 soccer clubs in Bangladesh. During follow‐up, all knee‐related injuries were recorded, including associated factors, and the number of ACL injuries was confirmed by magnetic resonance imaging (MRI). Incidence of ACL injury was compared by age group (11–14 years; 15–18 years; 19–22 years; and >22 years), sex (male and female), injury mechanism (contact‐person; contact‐friction; and overuse/non‐contact), and severity (Grade I/II/III; pain intensity—Visual Analog Scale: VAS).

**Results:**

A total of 110 MRI‐confirmed ACL injuries were recorded, with an overall incidence rate of 0.16 per 1000 exposure hours (95% CI: 0.133–0.195) and a cumulative proportion of 5.94% (95% CI: 4.90–7.11). Incidence increased progressively with age, from 0.05 in 11–14 years to 0.23 in > 22 years (*p* < 0.001). Females showed a higher crude incidence than males (0.20 vs. 0.14 per 1000 h; *p* = 0.041), though this was not significant after adjustment. Contact mechanisms predominated (0.07 player‐to‐player, 0.06 surface‐related) compared with non‐contact injuries (0.03; *p* = 0.008). By severity, Grade II injuries were most frequent (0.09 per 1000 h).

**Conclusion:**

This study indicates a higher incidence of ACL injuries among Bangladeshi soccer players, supported by subgroup analyses. It examines key associated factors, including age, sex, injury mechanism, and severity. These findings should be interpreted with caution and not considered definitive. Overall, they may help guide targeted prevention strategies.

## Summary

What Is Already Known on This Topic

ACL injury is among the most common and serious sports injuries in soccer players, with age, sex, and rotational knee injury mechanism recognized as key risk factors worldwide. However, little is known about its occurrence and contributing factors in South Asian countries, such as Bangladesh, where organized injury surveillance and prevention efforts are limited.

What This Study Adds

This study provides the first prospective data on ACL injuries among Bangladeshi soccer players, indicating a higher incidence compared with international reports. It also identifies age, sex, and contact‐related mechanisms as key contributing factors, which may show some variation from established global patterns. These findings should be interpreted with caution and not in a definitive or overgeneralized manner.

How This Study Might Affect Research, Practice, Or Policy

The findings establish a foundation for future research on long‐term outcomes and targeted prevention strategies in Bangladeshi soccer players. It also emphasizes the need for policy investment in safer playing environments, structured rehabilitation, and nationwide implementation of evidence‐based training programs.

## 1 Introduction

A common and debilitating injury among athletes is anterior cruciate ligament (ACL) injury. It has a relatively high incidence, especially among athletes such as soccer players, and can occur through contact or non‐contact mechanisms [1, 2]. The ACL restricts anterior movement of the tibia relative to the femur. It extends from the posterior medial aspect of the lateral femoral condyle in the intercondylar notch to the anterior part of the tibial intercondylar eminence [3]. Consequently, it is the most frequently injured knee ligament in sports. ACL tears can result from contact or non‐contact injuries, often happening when an athlete hyperextends the knee or quickly changes direction while moving [2]. Participation in high‐intensity and competitive sports increases the risk of ACL injuries. Therefore, ACL rupture is among the most serious injuries in soccer, potentially causing long recovery times or retirement, as well as long‐term health issues such as degenerative joint disease [2, 4].

A study found that national soccer players with ACL injuries incurred losses exceeding $2 million compared to players without such injuries [5]. The physical demands of soccer, especially rotational movements involving knee flexion and cutting, make athletes more susceptible [2]. Females face a two‐ to eightfold higher risk of ACL injury compared to males performing the same activities [6]. Soccer shows a higher incidence of ACL injuries among female athletes (1.5 injuries per 100 athletic exposures) and ranks third among the highest incidences in male players. Despite this growing body of literature, most epidemiological data originate from high‐income countries, particularly in North America and Europe, where sports infrastructure, training practices, injury surveillance systems, and preventive strategies are well established [7, 8].

In contrast, prospective epidemiological data from South Asian countries, including Bangladesh, are notably scarce, and the sporting context differs considerably. Soccer in Bangladesh is marked by variable access to professional training facilities, limited implementation of structured injury‐prevention programs, inconsistent medical and physiotherapy support, and differing patterns of play, including variations in playing surfaces and competitive intensity. Furthermore, cultural and socioeconomic factors may influence training exposure, injury reporting behaviors, and return‐to‐play decisions [3, 7]. These contextual differences may alter both the incidence and patterns of ACL injuries, making it inappropriate to generalize findings from Western populations to South Asian athletes.

The lack of robust, context‐specific data presents a significant barrier to developing effective, evidence‐based injury prevention and management strategies tailored to this population. Understanding the epidemiology of ACL injuries among Bangladeshi soccer players is therefore essential for informing clinicians, coaches, and policymakers and for guiding the implementation of targeted risk‐reduction programs. In particular, subgroup analyses by age, sex, injury mechanism, and injury severity are crucial for identifying high‐risk groups and modifiable factors in this unique sporting environment.

Therefore, this study aimed to determine the incidence of anterior cruciate ligament injuries among 1853 Bangladeshi soccer players over a 1‐year period, with subgroup analyses according to age, sex, injury mechanism, and severity. It is hypothesized that ACL injury incidence will vary significantly across these subgroups, with higher rates observed among female players, older age groups, specific injury mechanisms (both contact and non‐contact), and more severe injury grades. By addressing the current gap in South Asian sports injury epidemiology, this prospective cohort study seeks to provide foundational evidence to support the development of population‐specific injury prevention and management strategies.

## 2 Methods

### 2.1 Design and Ethics

A 1‐year prospective cohort study was conducted between July 2024 and June 2025 among 1853 soccer players from 12 clubs in Bangladesh. Ethical approval was received from the Institute of Physiotherapy Rehabilitation and Research of the Bangladesh Physiotherapy Association (BPA) and the sports‐related ethical board in Bangladesh (reference number: BPA‐IPRR/IRB/2024/05/378) on April 21, 2024. All participants and their legal guardians provided written informed consent. This report follows the Strengthening the Reporting of Observational Studies in Epidemiology for Sports Injury and Illness Surveillance (STROBE‐SIIS) guidelines [9].

### 2.2 Sample, Patient, and Public Involvement

**Figure 1:**
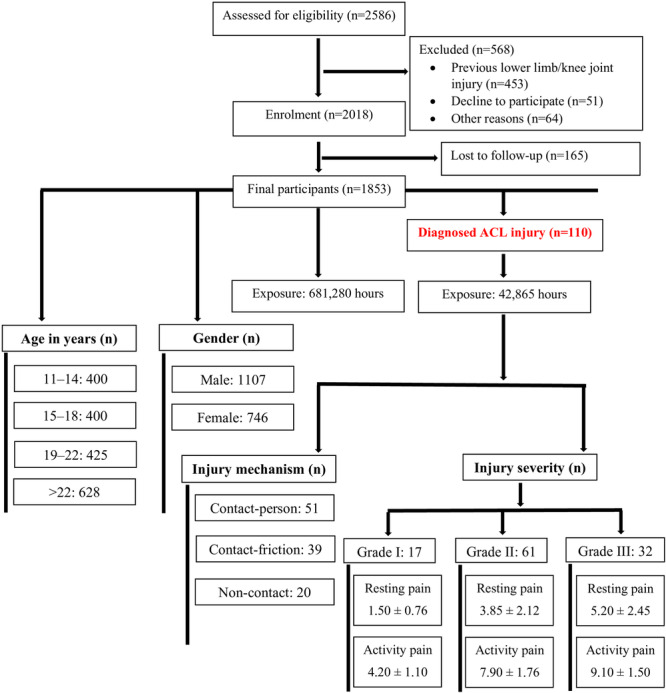
Study overview.

This study involved 1853 registered soccer players from 12 professional and semi‐professional clubs in Bangladesh, using a cluster‐based sampling approach, in which all eligible registered players within each club were invited to participate in this prospective cohort study. The included participants primarily represented competitive professional and semi‐professional levels, with regular engagement in structured training sessions and competitive matches throughout the study period. This approach enabled comprehensive exposure tracking and minimized selection bias within clubs. Initially, 2018 players were enrolled in this study. However, 165 players were lost to follow‐up. Finally, we completed this study with 1853 players. Participation required players to be actively involved in regular training and competitive matches during the study period. Players with pre‐existing severe knee injuries and other conditions that may interfere with our study findings were also excluded. Recruitment was conducted directly through the clubs with the help of responsible research assistants. Club officials and team coaches, who received thorough briefings on the study objectives, procedures, and ethical considerations during a pre‐study orientation, facilitated communication with the players. After being informed about the study, players and their legal guardians who provided written consent were enrolled. Players and coaches were actively involved during the study design phase to ensure practical applicability. Specifically, their input contributed to: (i) aligning data collection schedules with existing training and competition calendars, which minimized disruption and improved participant retention (feasibility); (ii) developing standardized and easily understandable injury reporting formats, including simplified definitions and reporting timelines, which reduced reporting errors and enhanced consistency across clubs (reliability); and (iii) adapting injury surveillance procedures to reflect local playing conditions, such as variations in pitch quality, training intensity, and medical support availability, thereby ensuring that the data accurately represented the real‐world soccer context in Bangladesh (contextual relevance). Furthermore, player feedback led to the incorporation of user‐friendly reporting methods, such as simplified injury logs and regular follow‐up reminders, which improved compliance and the completeness of injury data. Figure 1 provides an overview of this study.

### 2.3 Baseline Assessment

At the beginning of the study, a trained research team collected baseline data from all participating soccer players to identify potential risk factors for ACL injury. Variables included age (years), sex, playing position, regular training, frequency and hours of competitive match exposure, and history of previous knee or lower‐limb injuries. Previous injury history was obtained through structured interviews and, when available, confirmed with club medical records. This assessment was conducted under standardized conditions at each club to ensure consistency among participants. These baseline assessments provided reliable reference data for subgroup analyses of ACL injury incidence by age, sex, injury mechanism, and severity over the 1‐year follow‐up.

### 2.4 Observational Period

The study was carried out over a 1‐year follow‐up period (July 2024–June 2025), during which all participating soccer players were prospectively monitored. Exposure was defined as participation in training sessions and competitive matches, recorded weekly by team coaches and research assistants using a standardized log sheet. In the event of an injury, the coach, medical staff, and research team completed a standardized injury report, capturing details about the context (training or match), the specific injury mechanism (contact‐person, contact‐friction, or non‐contact/overuse), severity, and pain intensity (Visual Analog Scale [VAS]). All suspected ACL injuries were referred for confirmation via magnetic resonance imaging (MRI) at designated diagnostic centers, where experienced radiologists interpreted the images. Based on the MRI report, injury severity (Grade I–III) was documented and confirmed. Logistical and financial support was maintained through collaboration with each club and the research team, in accordance with the agreements. The research monitoring team maintained ongoing contact with clubs and research assistants to ensure timely and accurate documentation of injuries. Data collection procedures adhered to the International Olympic Committee consensus guidelines for injury surveillance in sport, ensuring consistency and reliability [9].

### 2.5 Statistical Analysis

All statistical analyses were performed using IBM SPSS Statistics version 26.0 (IBM Corp., Armonk, NY, USA). Data were examined for completeness, consistency, plausibility, and distributional assumptions before analysis. Participant characteristics and injury‐related variables were summarized using descriptive statistics. Continuous variables are presented as mean ± standard deviation (SD), whereas categorical variables are reported as frequencies and percentages. The incidence of ACL injury was calculated as the number of injuries per 1000 exposure hours, with corresponding 95% confidence intervals (CIs). Total exposure time included both training and match participation. Cumulative incidence proportions were also calculated for each subgroup. Factors associated with ACL injury incidence were examined using Poisson regression models with a log‐link function. Prior to model fitting, the assumptions of the Poisson regression model were evaluated. Dispersion was assessed by examining the ratio of the Pearson chi‐square statistic to the residual degrees of freedom and deviance statistics. No evidence of substantial overdispersion was identified; therefore, the Poisson regression model was considered appropriate, and a negative binomial model was not required. To account for variations in exposure time, the natural logarithm of total exposure hours was incorporated as an offset term. Crude incidence rate ratios (IRRs) and adjusted IRRs with 95% CIs were estimated. Variables included in the multivariable model were age group, sex, and injury mechanism based on epidemiological relevance and prior evidence. Regression coefficients (*β*), standard errors (SE), Wald *χ* ^2^ statistics, adjusted p‐values, and IRRs were reported. Among athletes who sustained ACL injuries, injury severity, incidence characteristics, and pain outcomes were analyzed separately. Pain scores at rest and during activity were compared across injury severity grades (Grade I–III) using one‐way analysis of variance (ANOVA). To identify factors independently associated with pain during activity, multiple linear regression analysis was conducted among injured athletes, with VAS pain during activity as the dependent variable. VAS pain at rest, exposure hours, and injury severity grade were entered simultaneously as independent variables. Regression coefficients (*β*), standard errors (SE), standardized coefficients (*β*), *t*‐values, and *p*‐values were reported. All statistical tests were two‐tailed, and a *p*‐value < 0.05 was considered statistically significant [10].

## 3 Results

**Figure 2:**
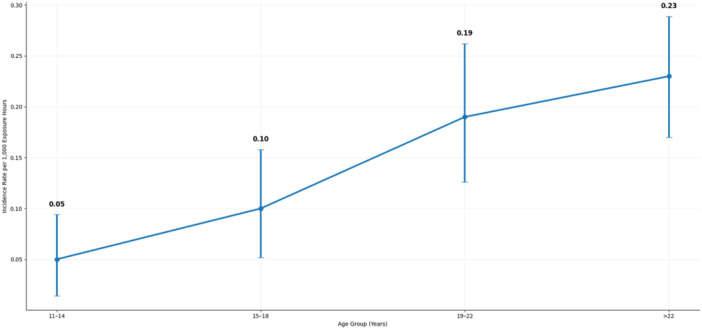
Age‐specific ACL injury incidence during 1‐year follow‐up.

**Figure 3:**
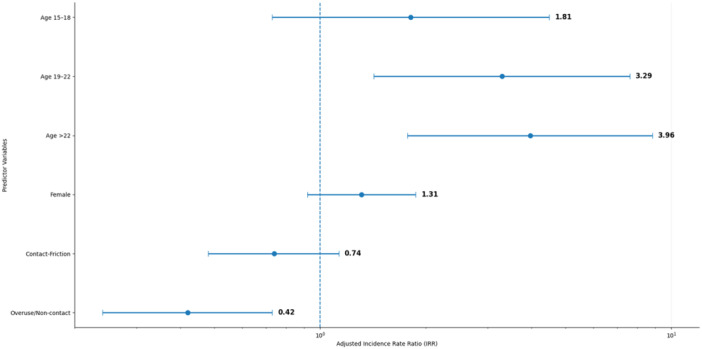
Adjusted incidence of ACL injury.

**Figure 4:**
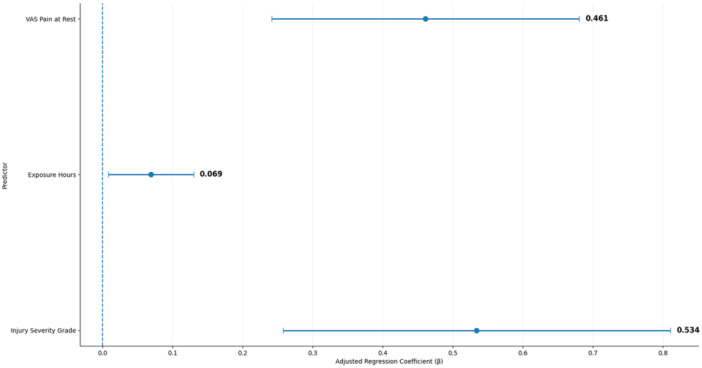
Factors associated with activity‐related pain.

A total of 1853 Bangladeshi soccer players were prospectively monitored over one competitive season, contributing 681,280 cumulative exposure hours from training and match participation. During follow‐up, 110 ACL injuries were identified, yielding an overall incidence rate of 0.16 injuries per 1000 exposure hours (95% CI: 0.133–0.195) and a cumulative incidence proportion of 5.94% (95% CI: 4.90%–7.11%). Detailed incidence estimates and subgroup analyses are presented in Tables 1, 2, 3, 4, while visualizations are provided in Figures 2, 3, 4.

**Table 1:** Incidence of ACL injury by demographic and mechanism factors.

Variable	Category	Players (*n*)	Total exposure (hours)	ACL injuries (*n*)	ACL injured exposure (hours)	Cumulative incidence proportion (%)	Incidence per 1000 h	95% CI	*p*‐value
Age (years)	11–14	400	129,600	7	2460	1.75	0.05	0.014–0.094	< 0.001
	15–18	400	143,200	15	5665	3.75	0.10	0.052–0.158	
	19–22	425	159,800	31	12,340	7.29	0.19	0.126–0.262	
	> 22	628	248,680	57	22,400	9.08	0.23	0.170–0.289	
Sex	Male	1107	436,710	61	25,500	5.51	0.14	0.105–0.175	0.041
	Female	746	244,570	49	17,365	6.57	0.20	0.144–0.256	
Injury mechanism	Contact‐person	—	—	51	18,000	2.75	0.07	0.054–0.096	0.008
	Contact‐friction	—	—	39	14,000	2.10	0.06	0.040–0.077	
	Overuse/Non‐contact	—	—	20	10,865	1.08	0.03	0.017–0.041	

*Note:* Incidence rates are expressed per 1000 exposure hours. Exposure hours included training and match participation. Statistical significance was defined as *p* < 0.05.

Abbreviations: ACL, anterior cruciate ligament; CI, confidence interval.

**Table 2:** Multivariable Poisson regression analysis of factors associated with ACL injury.

Variable	Category	Crude IRR (95% CI)	Adjusted IRR (95% CI)	*β* Coefficient	Standard error	Wald *χ* ^2^	Adjusted *p*‐value
Age group (years): 11–14, Ref.	15–18	1.94 (0.79–4.73)	1.81 (0.73–4.49)	0.59	0.46	1.66	0.197
	19–22	3.58 (1.56–8.18)	3.29 (1.42–7.61)	1.19	0.43	7.66	0.006
	> 22	4.23 (1.91–9.37)	3.96 (1.77–8.84)	1.38	0.41	11.34	< 0.001
Sex: Male, Ref.	Female	1.43 (1.01–2.02)	1.31 (0.92–1.87)	0.27	0.18	2.31	0.128
Injury mechanism: Contact‐person, Ref.	Contact‐friction	0.76 (0.50–1.15)	0.74 (0.48–1.13)	−0.30	0.22	1.85	0.174
	Overuse/non‐contact	0.39 (0.23–0.68)	0.42 (0.24–0.73)	−0.87	0.28	9.64	0.002

*Note:* Estimates were derived from Poisson regression models with a log‐link function and log‐transformed exposure hours included as an offset term. Adjusted models simultaneously included age group, sex, and injury mechanism. Statistical significance was defined as *p* < 0.05.

Abbreviations: CI, confidence interval; IRR, incidence rate ratio; Ref., reference category.

**Table 3:** Injury severity, incidence, and pain characteristics.

Severity (grade)	ACL injuries (*n*)	Exposure (hours)	Cumulative incidence proportion (%)	Incidence per 1000 h	95% CI	*p*‐value	VAS pain at rest (mean ± SD)	VAS Pain during activity (mean ± SD)
Grade I	17	7468	15.45	0.02	0.013–0.038	< 0.001	1.50 ± 0.76	4.20 ± 1.10
Grade II	61	22,562	55.45	0.09	0.068–0.113		3.85 ± 2.12	7.90 ± 1.76
Grade III	32	12,835	29.09	0.05	0.031–0.063		5.20 ± 2.45	9.10 ± 1.50
Overall *p*‐value (pain)							< 0.001	< 0.001

*Note:* Incidence rates are expressed per 1000 exposure hours. Exposure hours included training and match participation. Statistical significance was defined as *p* < 0.05.

Abbreviations: ACL, anterior cruciate ligament; CI, confidence interval.

**Table 4:** Multivariable linear regression analysis of factors associated with pain during activity.

Variable	*β* Coefficient	Standard error	Standardized *β*	*t*‐value	*p*‐value
VAS pain at rest	0.461	0.112	0.382	4.12	< 0.001
Exposure hours	0.069	0.031	0.187	2.23	0.026
Injury severity grade	0.534	0.141	0.351	3.79	< 0.001

*Note:* Multiple linear regression was performed with VAS pain during activity as the dependent variable. *β* coefficients represent adjusted regression estimates. All variables were entered simultaneously into the model.

Statistical significance was defined as *p* < 0.05.

### 3.1 Age‐Specific Incidence of ACL Injury

ACL injury incidence increased progressively across age categories (Table 1). Players aged > 22 years demonstrated the highest incidence rate (0.23 injuries per 1000 exposure hours; 95% CI: 0.170–0.289), followed by those aged 19–22 years (0.19 injuries per 1000 exposure hours; 95% CI: 0.126–0.262). In contrast, substantially lower rates were observed among players aged 15–18 years (0.10 injuries per 1000 exposure hours; 95% CI: 0.052–0.158) and 11–14 years (0.05 injuries per 1000 exposure hours; 95% CI: 0.014–0.094). Age‐related differences were statistically significant (*p* < 0.001), indicating a greater burden of ACL injury among older players.

### 3.2 Sex‐Specific Incidence of ACL Injury

Female players exhibited a significantly higher ACL injury incidence rate than male players (0.20 vs. 0.14 injuries per 1000 exposure hours, respectively; *p* = 0.041) (Table 1). Although females experienced a greater injury burden in unadjusted analyses, the magnitude of this association was attenuated after adjustment for potential confounders.

### 3.3 Injury Mechanism

Significant differences in ACL injury incidence were observed according to injury mechanism (*p* = 0.008) (Table 1). Contact‐related injuries accounted for the majority of ACL injuries, with player‐to‐player contact representing the highest incidence rate (0.07 injuries per 1000 exposure hours; 95% CI: 0.054–0.096), followed by friction/contact with the playing surface (0.06 injuries per 1000 exposure hours; 95% CI: 0.040–0.077). Non‐contact/overuse mechanisms demonstrated the lowest incidence rate (0.03 injuries per 1000 exposure hours; 95% CI: 0.017–0.041).

### 3.4 Injury Severity and Pain Characteristics

Among the 110 ACL injuries, Grade II injuries were the most common, accounting for 55.5% of all cases, followed by Grade III (29.1%) and Grade I injuries (15.5%) (Table 3). Incidence rates were highest for Grade II injuries (0.09 injuries per 1000 exposure hours; 95% CI: 0.068–0.113). Pain severity increased significantly with injury grade. Mean VAS pain scores at rest increased from 1.50 ± 0.76 in Grade I injuries to 5.20 ± 2.45 in Grade III injuries (*p* < 0.001), while VAS pain during activity increased from 4.20 ± 1.10 to 9.10 ± 1.50 across the same severity spectrum (*p* < 0.001), demonstrating a clear dose–response relationship between injury severity and symptom intensity.

### 3.5 Multivariable Predictors of ACL Injury

Results from the multivariable Poisson regression model are presented in Table 2. After adjustment for sex and injury mechanism, players aged 19–22 years (adjusted incidence rate ratio [aIRR] = 3.29, 95% CI: 1.42–7.61; *p* = 0.006) and > 22 years (aIRR = 3.96, 95% CI: 1.77–8.84; *p* < 0.001) remained at significantly greater risk of ACL injury compared with players aged 11–14 years. Female sex was not independently associated with ACL injury risk after adjustment (aIRR = 1.31, 95% CI: 0.92–1.87; *p* = 0.128). In contrast, non‐contact/overuse mechanisms were independently associated with a lower injury incidence relative to player‐to‐player contact injuries (aIRR = 0.42, 95% CI: 0.24–0.73; *p* = 0.002).

### 3.6 Factors Associated With Pain During Activity

Multivariable linear regression analysis identified VAS pain at rest (*β* = 0.461, *p* < 0.001), injury severity grade (*β* = 0.534, *p* < 0.001), and exposure hours (*β* = 0.069, *p* = 0.026) as significant independent predictors of pain during activity (Table 4). Injury severity demonstrated the strongest association with activity‐related pain, indicating that greater structural damage was accompanied by substantially higher symptom burden.

### 3.7 Data Quality and Reliability

Exposure hours and injury events were prospectively documented using a standardized surveillance protocol across all participating clubs. Prior to study commencement, team coaches and research assistants received standardized training on surveillance procedures, injury definitions, and data recording to ensure consistent implementation. Exposure data were independently recorded by team coaches immediately after each training session and match using standardized exposure logs and were subsequently verified weekly by trained research assistants. Throughout the study period, the coordinating research team conducted regular monitoring visits and periodic audits to evaluate protocol adherence, data completeness, and consistency across participating clubs. Any discrepancies between exposure records, injury reports, and available medical documentation were resolved through review of the original records and direct communication with coaches, research assistants, and participants before database finalization. Injury data were additionally cross‐checked against available club medical records, and all suspected ACL injuries were confirmed by MRI interpreted by experienced musculoskeletal radiologists, minimizing the risk of outcome misclassification. Missing or inconsistent entries were resolved promptly through predefined data verification procedures, resulting in a high level of data completeness (> 95%). Given the minimal extent of missing data, complete‐case analysis was performed. Collectively, these standardized surveillance, verification, and quality assurance procedures strengthened the accuracy, completeness, and internal validity of the study data, although the findings should be interpreted within the inherent limitations of prospective observational sports injury surveillance.

## 4 Discussion

This prospective cohort study provides the first exposure‐adjusted epidemiological data on ACL injuries among Bangladeshi soccer players. The prevalence of ACL injuries was significant, with older age and injury mechanism independently linked to the occurrence of injuries, but the elevated crude incidence seen among female athletes was not sustained following multivariable correction. These results provide significant context‐specific information from an environment where prospective sports injury monitoring is scarce and establish a basis for future preventive research.

The observed ACL injury incidence falls within the upper range of estimates reported in previous prospective soccer cohorts; however, direct comparisons should be made with caution due to substantial heterogeneity across studies in participant characteristics, injury definitions, exposure measurement, competition level, surveillance methods, and diagnostic confirmation procedures. Accordingly, the present findings primarily reflect the ACL injury burden within this Bangladeshi cohort rather than indicating an inherently higher or lower risk compared with global reports. Although factors such as neuromuscular training exposure, training load, playing surface conditions, environmental influences, and access to sports medicine services have been previously implicated in ACL injury risk, these variables were not specifically examined in the current study [4, 11, 12, 13]. Therefore, their potential contribution to the observed incidence should be interpreted as exploratory hypotheses requiring further investigation rather than as confirmed explanatory determinants within the context of this study.

Age was a significant independent predictor of ACL injury. The prevalence of injuries grew with age, peaking among the oldest participants. Multivariable Poisson regression indicated that athletes in older age cohorts had a significantly elevated risk of ACL injury compared to the youngest players, with an incidence increase of about three to four times. These results align with longitudinal and registry‐based research indicating an increased risk of ACL injury between late adolescence and early adulthood [4, 13]. This phase is marked by heightened engagement in high‐intensity competition, augmented training loads, and increasing exposure to high‐risk game scenarios. While physical maturation improves strength and athletic performance, neuromuscular control may not develop in parallel, leading to inefficient movement patterns and increased ligament stress during dynamic sports activities [11, 13].

Moreover, cumulative exposure to repetitive mechanical loading may contribute to fatigue‐related impairments in motor control and joint stabilization, thereby increasing susceptibility to injury [12] The absence of a statistically significant adjusted association in the younger age groups suggests that age itself may not be the sole determinant of risk; rather, the transition to higher competitive levels and greater exposure volumes may represent critical thresholds for ACL injury occurrence. These findings support the implementation of age‐targeted injury prevention strategies, particularly among older adolescents and adults in soccer [4, 11].

Female athletes demonstrated a greater crude incidence of ACL injuries compared to their male counterparts; however, this correlation diminished and became statistically insignificant after adjustment for age and injury mechanism, indicating that the noted sex disparity was partially attributable to variations in exposure and other contextual factors. These results highlight the need to consider possible confounding factors when assessing sex‐specific injury risk [11, 13]. While female sex is acknowledged as a significant risk factor for ACL injury, attributed to anatomical, hormonal, and neuromuscular disparities, the extent of this correlation differs among sports and populations, influenced by training exposure, competitive demands, and environmental context [12, 14, 15, 16]. Thus, our results should not be seen as contradicting the established data, but rather as suggesting that the independent impact of sex may be diminished after adjusting for pertinent factors within this cohort. The association's direction remained aligned with prior studies, endorsing the ongoing use of sex‐specific ACL injury prevention techniques.

A significant discovery was the prevalence of contact‐related ACL injuries, with player‐to‐player contact representing the greatest frequency, whereas non‐contact and overuse causes were relatively rare and linked to a lower adjusted injury risk. This tendency differs from findings in top soccer groups, where non‐contact processes usually prevail [17, 18]; nonetheless, such discrepancies should be viewed with caution. They may indicate disparities in playing level, pitch quality, competition intensity, refereeing, technical competence, and contextual factors rather than intrinsic differences in ACL injury processes [17, 18, 19, 20]. The findings indicate that the contribution of contact and non‐contact injuries may vary by context and emphasize the need to augment athlete‐centered neuromuscular prevention programs with comprehensive strategies to ensure safe play, coaching methodologies, and playing environments, especially in contexts where contact‐related injuries are more common.

Analysis of injury severity indicated that Grade II ACL injuries were the most prevalent, including more than 50% of cases, followed by Grade III and Grade I injuries. This distribution aligns with previous epidemiological literature indicating a prevalence of partial ligament injuries in athletic populations; however, direct comparisons should be approached cautiously due to variability in diagnostic criteria, imaging accessibility, and healthcare‐seeking behaviors across studies [21, 22, 23]. The use of MRI‐validated diagnoses in this research strengthens the reliability of severity categorization and reduces the likelihood of misclassification bias. The level of pain demonstrated a distinct dose–response relationship across injury grades, as both resting and activity‐related VAS ratings escalated steadily from Grade I to Grade III injuries, indicating a heightened symptom load with increased structural damage. Regression analysis consistently indicated injury severity as the most significant independent predictor of pain during exercise, followed by pain at rest and total exposure hours. These findings are physiologically realistic and align with documented pathways whereby greater ligament injury leads to increased nociceptive input, inflammatory activity, and functional joint instability, thereby exacerbating movement‐related discomfort [24]. The observed correlation between structural damage severity and symptom intensity highlights the need to combine anatomical and clinical evaluations when measuring injury burden and informing rehabilitation approaches.

## 5 Strengths and Limitations

This study possesses several important strengths, including its prospective cohort design, large sample size, and comprehensive exposure assessment, which enabled accurate estimation of incidence rates using person‐time denominators. The use of MRI‐confirmed diagnoses minimized outcome misclassification and enhanced diagnostic validity. Furthermore, multivariable regression analyses allowed adjustment for important confounding factors, strengthening the internal validity of the findings.

Nevertheless, several limitations should be acknowledged. Residual confounding remains possible because potentially relevant variables, including playing position, previous ACL injury, neuromuscular performance, training intensity, and psychological factors, were not comprehensively measured. Although participants were recruited from 12 clubs, clustering by club was not explicitly accounted for in the regression analyses. Consequently, within‐club correlation may have resulted in modest underestimation of standard errors. Future multicenter studies should incorporate multilevel or mixed‐effects regression models to appropriately account for club‐level clustering. Although incidence rates facilitate comparison across studies, differences in surveillance methodologies and competition levels limit direct external comparability. Pain outcomes were assessed using self‐reported measures and may therefore be subject to reporting variability. Finally, as an observational study, causal relationships cannot be established.

Future studies should use multicenter designs with multilevel modeling to account for club‐level clustering and incorporate key intrinsic and extrinsic risk factors, including neuromuscular, biomechanical, training load, positional, and psychological variables, while also evaluating the effectiveness of ACL injury prevention programs in real‐world competitive settings.

## 6 Implications

The current findings have significant implications for clinicians, coaches, sports organizations, and policymakers, and should be interpreted with caution given the study's observational design. The documented injury burden underscores the need for the systematic use of evidence‐based ACL injury prevention techniques, especially neuromuscular training programs focused on movement control, balance, landing mechanics, and dynamic knee stability. Given the elevated risk observed among older and young adults, preventive initiatives should be prioritized for these groups. The prevalence of contact‐related injuries suggests that comprehensive strategies—possibly including improvements in playing conditions, officiating protocols, coaching methodologies, and player education—could help mitigate risk, although causal relationships cannot be inferred from the current data. Enhancing access to prompt diagnosis, imaging, and structured rehabilitation pathways may improve clinical outcomes and reduce long‐term impairment. The establishment of national injury monitoring systems and sport‐specific injury prevention frameworks may serve as a crucial basis for evidence‐based policy and athlete safety efforts.

## 7 Conclusion

This prospective cohort study demonstrated that ACL injuries represent a substantial burden among Bangladeshi soccer players. Increasing age and injury mechanism were identified as independent predictors of ACL injury, whereas the higher crude incidence observed among female players was not maintained after adjustment for confounding factors. Contact‐related mechanisms, particularly player‐to‐player contact, accounted for the largest proportion of injuries, suggesting a context‐specific injury profile influenced by environmental and organizational conditions. Grade II injuries were most prevalent, and pain severity increased progressively with injury grade. Collectively, these findings underscore the importance of targeted injury‐prevention strategies, strengthened injury surveillance, optimized playing environments, and evidence‐based rehabilitation programs to help reduce the ACL injury burden and improve athlete health outcomes in Bangladesh and comparable sporting contexts.

## Author Contributions

**Sohraf Hossain:** conceptualization, validation, data curation, formal analysis, writing – original draft, writing – review and editing, methodology, project administration. **Kazi Md. Azman Hossain:** methodology, software, formal analysis, investigation, visualization, writing – original draft, writing – review and editing, project administration. **Md. Shafiqul Islam:** methodology, investigation, resources, conceptualization, writing – review and editing, funding acquisition, project administration, software. **Md. Adnan Ibne Reza:** methodology, data curation, funding acquisition, writing – review and editing, resources. **Tamzid Hossain:** investigation, funding acquisition, supervision, writing – review and editing. **Md. Nazmul Haq:** supervision, validation, writing – review and editing. **Kulsum Akter:** writing – review and editing, writing – original draft, supervision, conceptualization, validation.

## Funding

The authors have nothing to report.

## Ethics Statement

Ethical approval was obtained from the Institute of Physiotherapy Rehabilitation and Research of the Bangladesh Physiotherapy Association (BPA) and the sports‐related ethical board in Bangladesh (reference number: BPA‐IPRR/IRB/2024/05/378). Before enrollment and publication, written and verbal informed consent were obtained from all the participants and/or their legal guardians.

## Consent

All participants and/or their legal guardians gave written informed consent prior to participation and publication.

## Conflicts of Interest

The authors declare no conflicts of interest.

## Patient and Public Involvement

Coaches and players actively contributed to the design and execution of this study. Their feedback guided the data collection and improved its contextual relevance. Details are in the Methods section.

## Provenance and Peer Review

Not commissioned; externally peer reviewed.

## Transparency Statement

The corresponding author, Kulsum Akter, affirms that this manuscript is an honest, accurate, and transparent account of the study being reported; that no important aspects of the study have been omitted; and that any discrepancies from the study as planned (and, if relevant, registered) have been explained.

## Data Availability

Data collected and analyzed in this study on the incidence of ACL injuries among Bangladeshi soccer players are available from the corresponding author upon reasonable request.
